# Identifying Patient-Reported Outcome Measure Documentation in Veterans Health Administration Chiropractic Clinic Notes: Natural Language Processing Analysis

**DOI:** 10.2196/66466

**Published:** 2025-04-02

**Authors:** Brian C Coleman, Kelsey L Corcoran, Cynthia A Brandt, Joseph L Goulet, Stephen L Luther, Anthony J Lisi

**Affiliations:** 1 Pain Research, Informatics, Multimorbidities, and Education Center, VA Connecticut Healthcare System, 950 Campbell Ave, West Haven, CT, 06516, United States, 1 2039325711; 2 Department of Emergency Medicine, Yale School of Medicine, Yale University, New Haven, CT, United States; 3 Department of Biomedical Informatics and Data Science, Yale School of Medicine, Yale University, New Haven, CT, United States; 4 Center of Innovation for Complex Chronic Healthcare, Edward Hines, Jr. VA Hospital, Hines, IL, United States; 5 College of Public Health, University of South Florida, Tampa, FL, United States

**Keywords:** Veterans Health Administration, natural language processing, quality of health care, chiropractic, patient reported outcome measures, NLP, AI, artificial intelligence, veteran, chiropractor, integrated health cohort, musculoskeletal diagnosis, musculoskeletal, quality of care, care, PROM, neural network, chiropractic care

## Abstract

**Background:**

The use of patient-reported outcome measures (PROMs) is an expected component of high-quality, measurement-based chiropractic care. The largest health care system offering integrated chiropractic care is the Veterans Health Administration (VHA). Challenges limit monitoring PROM use as a care quality metric at a national scale in the VHA. Structured data are unavailable, with PROMs often embedded within clinic text notes as unstructured data requiring time-intensive, peer-conducted chart review for evaluation. Natural language processing (NLP) of clinic text notes is one promising solution to extracting care quality data from unstructured text.

**Objective:**

This study aims to test NLP approaches to identify PROMs documented in VHA chiropractic text notes.

**Methods:**

VHA chiropractic notes from October 1, 2017, to September 30, 2020, were obtained from the VHA Musculoskeletal Diagnosis/Complementary and Integrative Health Cohort. A rule-based NLP model built using medspaCy and spaCy was evaluated on text matching and note categorization tasks. SpaCy was used to build bag-of-words, convoluted neural networks, and ensemble models for note categorization. Performance metrics for each model and task included precision, recall, and F-measure. Cross-validation was used to validate performance metric estimates for the statistical and machine-learning models.

**Results:**

Our sample included 377,213 visit notes from 56,628 patients. The rule-based model performance was good for soft-boundary text-matching (precision=81.1%, recall=96.7%, and F-measure=88.2%) and excellent for note categorization (precision=90.3%, recall=99.5%, and F-measure=94.7%). Cross-validation performance of the statistical and machine learning models for the note categorization task was very good overall, but lower than rule-based model performance. The overall prevalence of PROM documentation was low (17.0%).

**Conclusions:**

We evaluated multiple NLP methods across a series of tasks, with optimal performance achieved using a rule-based method. By leveraging NLP approaches, we can overcome the challenges posed by unstructured clinical text notes to track documented PROM use. Overall documented use of PROMs in chiropractic notes was low and highlights a potential for quality improvement. This work represents a methodological advancement in the identification and monitoring of documented use of PROMs to ensure consistent, high-quality chiropractic care for veterans.

## Introduction

Patient-reported outcome measures (PROMs) are standardized, validated questionnaires completed by patients to identify and quantify their perceptions of their health status [1]. These measures are often of interest to clinicians to assess condition severity and response to treatment as a component of a measurement-based care approach. Measurement-based care is a recommended practice in the management of musculoskeletal pain conditions [2], where biomarkers of disease severity are lacking and baseline and serial reassessment for progress may influence clinical decision-making. Using PROMs can improve communication and shared decision-making between patients and clinicians, enable contextualization of pain within a patient’s life, and may positively influence health and pain status [3].

In the Veterans Health Administration (VHA), patients with musculoskeletal pain may receive chiropractic care, where the use of PROMs is an expected component of high-quality care [4,5]. The VHA Office of Specialty Care Ongoing Professional Practice Evaluation quality program for chiropractors includes a quality metric stating “appropriate pain, functional, and/or other measures are documented and used to inform clinical decision making.” However, substantial challenges limit monitoring this important metric of high-quality care at a national scale. Digital data systems integration is limited for both remote and point-of-care data collection that may otherwise facilitate structured data collection, thus any PROM documentation often occurs in unstructured clinic notes. Quality evaluation of these notes typically requires time-intensive, peer-conducted chart review, with substantial human effort limiting ongoing monitoring [6].

The VHA Chiropractic Program has expanded rapidly in recent years driven by policy change and natural growth [7,8], with 299 facilities now offering on-site chiropractic care in fiscal year (FY) 2024, up 344% from 87 facilities in FY2017. Scalable solutions are needed to monitor established care quality metrics, such as PROM use, and ensure high-quality chiropractic care delivery across the enterprise. Further, such solutions could also be widely applicable to other disciplines managing musculoskeletal pain, which is highly prevalent and burdensome in the VHA system and beyond.

Natural language processing (NLP) of clinic text notes is one promising solution to extracting care quality data from unstructured text [9], with previous studies focusing on pain care quality in primary care and chiropractic care settings [10,11]. Use cases for NLP across other clinical domains highlight its potential utility in information extraction and analysis tasks. NLP methods have been used to accurately identify functional status impairment for patients with dementia based on electronic health record (EHR) clinic notes [12]. Additionally, NLP techniques have shown utility in information extraction, classification, and risk prediction tasks for patient-reported outcomes among cancer patients [13]. Relevant to pain management, NLP has been used to identify opioid use and misuse in clinic notes [14] and support decision support systems to impact clinical care [15]. More recently, the use of large-language models in data mining of EHR data, including clinical text data, has demonstrated promise and efficiency in information extraction and content analysis [16].

The objective of this study was to develop an NLP approach to identify PROMs documented in VHA chiropractic clinic text notes. We aimed to iteratively develop a rule-based pipeline and evaluated performance for a text span pattern matching task and a note categorization task. We also aimed to compare the performance of the rule-based method to statistical and machine learning methods for the note categorization task and estimate the overall prevalence of documented PROM use in the corpus. We hypothesize a rule-based NLP approach, when iteratively developed and refined, will demonstrate comparable performance to statistical and machine learning methods in identifying and categorizing PROM documentation within VHA chiropractic clinic text notes.

## Methods

### Study Setting, Data Sources, and Cohort

We conducted a secondary, retrospective analysis of the Musculoskeletal Diagnosis/Complementary and Integrative Health (MSD/CIH) Cohort [17]—an EHR data cohort of VHA patients receiving VHA health care for musculoskeletal conditions, with updated cohort entry through September 30, 2020. Study reporting was informed by published recommendations for reporting machine learning and NLP studies [18,19].

We identified patients in the MSD/CIH Cohort who received VHA chiropractic care at a VHA facility between October 1, 2017, and September 30, 2020. All chiropractic clinic visits were identified using an administrative clinic identifier denoting “Chiropractic Care.” Initiating chiropractic care in the VHA most often requires the placement of a referral order for consultation, with the consultation visit linked to this order in the EHR. We included only patients with an initial consultation visit during the study period and included all visits occurring within 1 year of their first consultation visit date. Population demographic and clinical characteristics were extracted from the EHR for each patient included in the sample, including age at first chiropractic consultation, sex of record, race/ethnicity, marital status, smoking status, service-connected disability percentage, and BMI. *International Classification of Diseases, 10th Revision* (*ICD-10*) codes were also extracted for each visit, and flags were used to denote whether a visit included a low back pain diagnosis, neck pain diagnosis, other spinal pain diagnosis, or any other diagnosis.

We obtained all clinic text notes linked to the identified visits to build a corpus of clinic visit notes. We excluded note types related to telecommunications and administrative events (eg, appointment scheduling, secure messaging), including only notes describing chiropractic care in the ambulatory, in-hospital, or telehealth setting.

### Data Preparation

Multiple notes can be written to describe the same identified chiropractic visit; for example, a resident chiropractor note and an attending chiropractor note may each contain data relevant to a single visit. We concatenated all notes linked to the same unique visit identifier on the same date of service (regardless of note author) to create a 1-to-1 relationship between visits and clinic notes. A unique character set was used as a delimiter to separate individual notes. To evaluate the amount of text present across notes in the corpus, we quantified each note length using tokenization based on whitespace splitting and visualized the data using a histogram of token lengths. We compared tokenized lengths across different types of visits and across FYs through visual comparison, Kruskal-Wallis tests across all groups, and Dunn tests across group pairs with a significance level *α* of 0.05 and a Bonferroni correction for multiple comparisons.

Additional metadata about each chiropractic care visit was extracted from the EHR and written into a header for each note, offset from the rest of the note using a unique character series delimiter. This included a patient identifier, visit identifier, visit date, VHA facility identifier code, the date of the first consultation visit, and the visit number with the total number of visits within the year after consultation (eg, “visit 3 of 6”), the number of days since the previous visit (if applicable), and the number of days to the next visit (if applicable).

Visits were tagged into the following five exclusive categories using conditional logic: (1) first consultation visit, (2) final visit within 1 year, (3) visit immediately preceding a 60-day gap in care, (4) visit immediately following a 60-day gap in care, and (5) other intermediate visit. These categories were identified to stratify visits on having a higher likelihood of potential use of PROMs at the beginning or end of an episode of chiropractic care or before or after a gap in chiropractic care.

A nationally representative, random stratified sample of 300 notes was selected for human annotation to be used as an initial training set. The stratification approach was designed to select an approximately equal number of notes for each visit category and was randomly repeated until a maximum number of facilities was included in the training set (78 of 79, with 1 facility excluded due to very few notes included in the corpus).

### Corpus Annotation

Two study investigators (BCC and KLC) with VHA chiropractic care subject-matter expertise annotated the training corpus using eHost, a Java-based annotation tool [20]. Annotators identified and tagged spans of text referencing documented use of PROMs and assigned a span attribute for the specific type of PROM based on an a priori list (Multimedia Appendix 1). The initial seed list of PROMs was a sample of validated PROMs potentially used by chiropractors addressing pain and function, particularly for spinal conditions. An additional attribute class was included for tagging an unspecified PROM that was not included in the list, which was reviewed for inclusion during subsequent iterations. We excluded all versions of the Numerical Rating Scale and Visual Analog Scales as unidimensional measures that have been criticized as potentially limited clinical importance [21], especially when trying to measure a complex, multifaceted condition like musculoskeletal pain [22,23]. An annotation guide was developed and iteratively revised/validated using a preparatory random note sample from a separate corpus during an annotation pilot. Interannotator agreement (IAA) was high across 3 samples of pilot notes (n_1_=50 notes, IAA_1_=78.8%; n_2_=100 notes, IAA_2_=84.5%; and n_3_=100 notes, IAA_3_=86.1%).

Annotation of the initial training set (n=300 notes) was iteratively completed in 100-note batches. IAA remained high across the 3 iterations of annotating the initial training set (IAA_1_=71.7%, IAA_2_=81.2%, and IAA_3_=87.1%). Adjudication of disagreement was achieved through review and discussion between the annotators. A third-person adjudicator was available to provide a final adjudication decision in the event of unresolved disagreement, though this did not become necessary.

### Initial NLP Model Development

**Figure 1.**
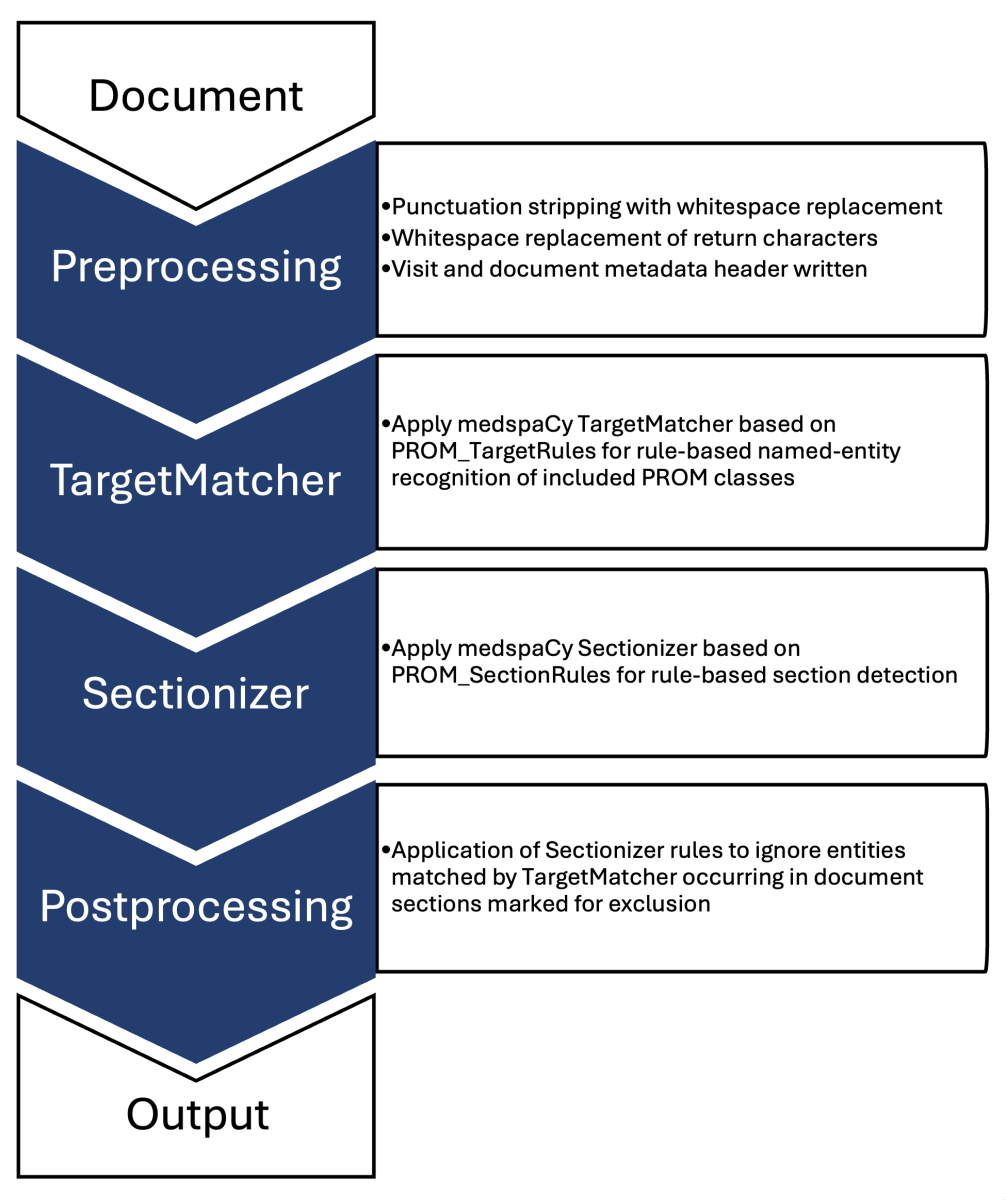
Rule-based natural language processing (NLP) medspaCy pipeline overview. PROM: patient-reported outcome measure.

A rule-based NLP pipeline (Figure 1) was built using medspaCy [24] (v1.0.0) and spaCy [25] (v3.6.0; Explosion) in Python (v3.8.5; Python Software Foundation). The initial pipeline used the medspaCy TargetMatcher and TargetRule functions to match spans of text in a spaCy Doc object. Matches were based on an initial set of rules to define text pattern rules for 15 predetermined PROMs, with an associated attribute referencing a specific measure assigned for each identified span.

For all iterations of NLP model development, evaluation statistics (precision, recall, and F-measure) were calculated based on true positives, false positives, and false negatives for three defined matching tasks: (1) strict-boundary matching, (2) soft-boundary matching, and (3) note categorization. Strict-boundary matching considered only the perfect overlap of the human annotation and NLP target matching methods to be a match in performance metric calculation. Soft-boundary matching was a fuzzy matching approach that allowed for flexibility in the overlap between the span start and span end positions of the human annotation and NLP target matching methods to define a match. Note categorization cast the results of the human annotation and NLP target matching to a binary document classification question, defining documented PROM use at the note level. All matching methods required matching on the assigned attribute for a specified PROM to be considered a match.

### NLP Model Refinement

The initial pipeline was run on the annotated first training set and false positives and false negatives were manually reviewed to revise the TargetMatcher rules and the overall NLP pipeline. Pipeline modifications were attentively designed to address only generalizable false positives and negatives and avoid overfitting the training data. During this review, false negatives were addressed by modifying TargetMatcher rules to better represent broader matching patterns (eg, abbreviations), supporting fuzzy matching (eg, typographic errors), and by adding TargetMatcher rules for 3 additional measures identified during annotation. False positives were addressed by adding sectionizer and postprocesser steps to the NLP pipeline. A medspaCy sectionizer component was added to define sections of the clinical note, with a Postprocessor component added to set rules for sections to ignore in defining PROM spans. This allowed the exclusion of inappropriate matches that were being identified in abbreviation lists, medication lists, goals of care, and other irrelevant sections of the note. The final rule-based model is available on GitHub [26].

After the development of an initial NLP rule-based model to identify PROM documentation, a second high-probability training set (n=200) was identified based on a preliminary model NLP output as programmatic labeling and annotated by a single investigator (BCC). This created a full annotation set of 500 notes and increased the prevalence of positive notes in the training set to balance the prediction problem.

Using the full training set, we trained an initial set of statistical and machine learning models to complete the note categorization task prediction and compared their performance to the rule-based model. The full annotated training set was randomly partitioned with 75% allocated to the model training set, 15% to the development set to tune hyperparameters, and 10% to the test set to evaluate performance. We tested 3 model architecture configurations built in spaCy: (1) a bag-of-words (BOW) model, (2) a convoluted neural network model, and (3) an ensemble model combining a linear BOW model and Tok2Vec model. Architecture configurations for each model are detailed in Multimedia Appendix 2 with source code available on GitHub [26]. All computational analysis was conducted using a secure virtual machine with a Windows 10 operating system, 8 virtual processors (x86-64, 2.60 GHz), and 16 GB memory, with variable overall runtime based on competing user demands.

### NLP Model Evaluation and Validation

For the initial evaluation of the rule-based model performance on the text matching and note categorization tasks, we evaluated performance based on precision, recall, and F-measure. To compare the performance of the rule-based model to the initial statistical and machine learning models for the note categorization task, precision, recall, F-measure, and accuracy were calculated. The area under the receiver operator characteristic curve (AUC-ROC) was calculated for the statistical and machine learning models. AUC-ROC was not calculated for the rule-based model as it is a deterministic model generating a binary decision output rather than a probability score output.

We used Monte Carlo and k-folds cross-validation (stratified and unstratified) to validate the calculated performance metrics for the initial statistical and machine learning models across multiple simulations. Monte Carlo cross-validation consisted of 100 simulation cycles randomly partitioning the annotated data set into 75% training/15% development/10% testing splits. k-folds cross-validation was performed using 10 cycles of 10-fold repeated cross-validation (100 total cycles), with and without stratification, and sampled 8 training folds, 1 development fold, and 1 test fold during each cycle. Precision, recall, F-measure, and AUC-ROC were calculated for each cycle across both cross-validation methods.

### Ethical Considerations

This study received exemption approval from the institutional review boards of the VA Connecticut Healthcare System (1690344-1) and Yale University (2000032830).

## Results

We identified 56,628 patients for inclusion in this study, with a total of 377,213 visits across the study period. Patient and visit characteristics are presented in Table 1. The patient population was consistent with those usually receiving VHA chiropractic care. Patients had a median of 5 (IQR 3-6) chiropractic care visits, with most visits occurring for low back pain. There were 14,198 patients (25.1%) who had at least one 60-day care gap. The tokenized text across the entire corpus had a mean length of 565 tokens (SD 434), with 4617 notes (1.2%) greater than 2000 tokens in length.

**Figure 2.**
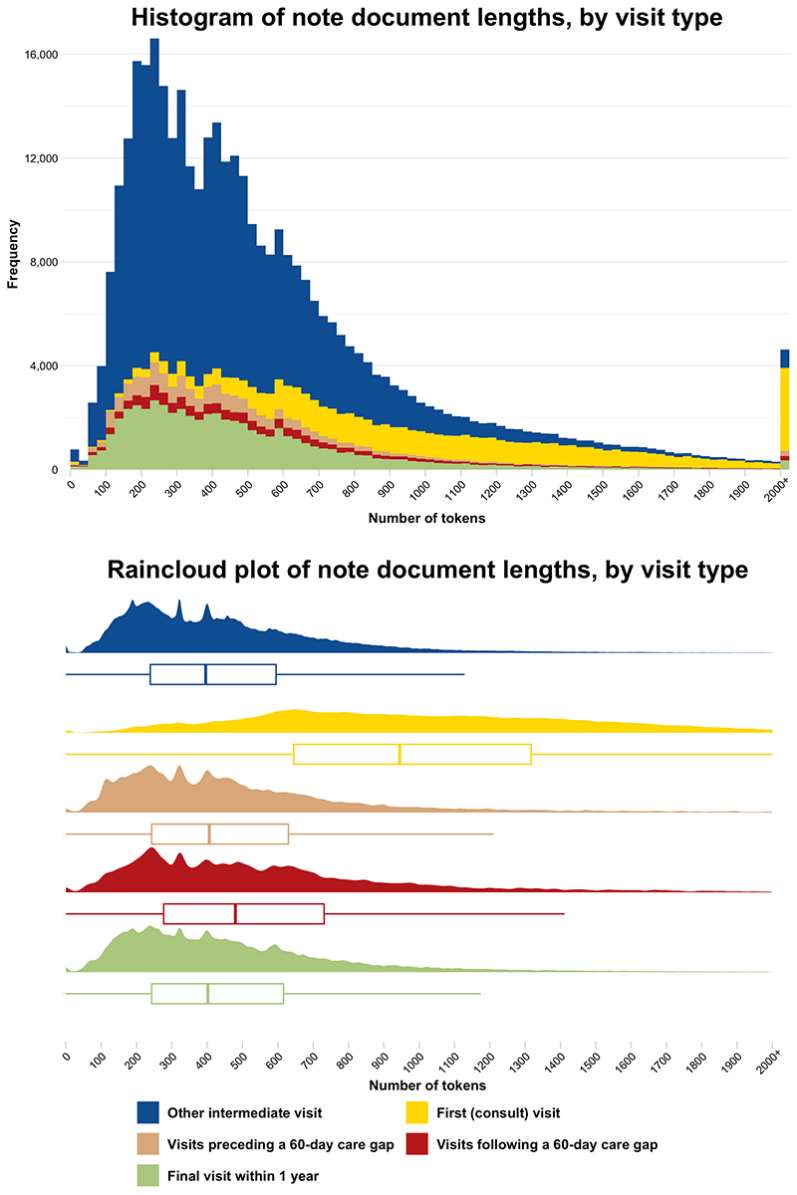
Tokenized text length of notes in the study corpus, split on whitespace characters, by visit type with an overflow bin for notes greater than 2000 tokens (n=4617).

**Figure 3.**
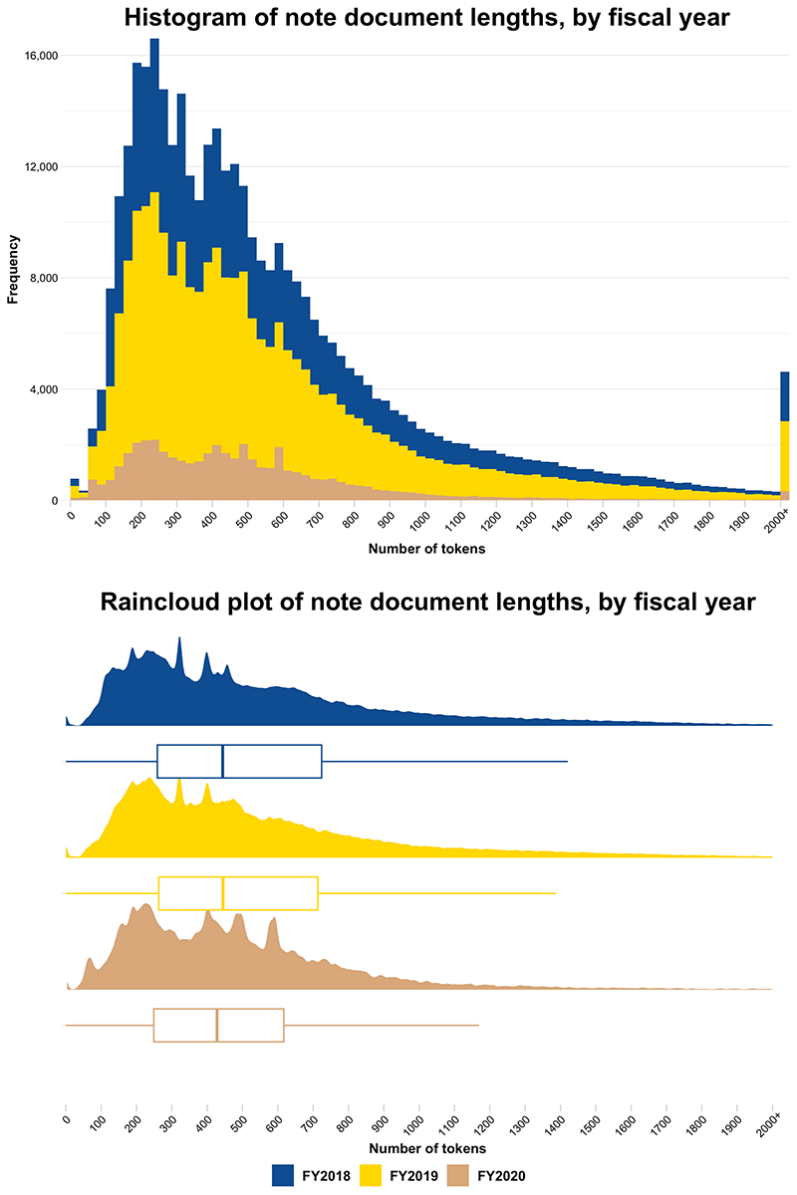
Tokenized text length of notes in the study corpus, split on whitespace characters, by fiscal year, with an overflow bin for notes greater than 2000 tokens (n=4617). FY: fiscal year.

Assessing the tokenized text lengths by visit type for the entire corpus split by whitespaces (Figure 2) showed a greater number of tokens in the first (consult) visits compared with the other visit types. The distribution of tokenized length of first (consult) visits was right-shifted compared with all other visit types, with a mean length of 1069 tokens (SD 565) and 3198 notes (5.6%) greater than 2000 tokens in length. The mean token length was 464 (SD 316) for other intermediate visits, 514 (SD 404) for visits preceding a 60-day care gap, 587 (SD 427) for visits following a 60-day care gap, and 490 (SD 358) for final visits within 1 year. A Kruskal-Wallis test demonstrated statistically significant differences across the 5 visit types (*P*<.001), with all corrected pairwise comparisons significant at *P*<.001 except visits preceding a 60-day care gap compared with final visits within 1 year (*P*=.008). Tokenized text lengths by FY (Figure 3) showed a mean length of 579 (SD 456) tokens in FY2018, 571 (SD 435) tokens in FY2019, and 496 (SD 348) tokens in FY2020. A Kruskal-Wallis test showed a statistically significant difference across the FY groups (*P*<.001), with corrected pairwise comparisons demonstrating significant differences between FY2020 and each of FY2018 and FY2019 (*P*<.001), but not between FY2018 and FY2019 (*P*≥.99).

For each included PROM, the text span match frequency and note categorization frequency between human annotation and the rule-based model output on the full training set and in the full-text corpus are presented in Multimedia Appendix 1. When the rule-based model was run on the full note corpus, there were 112,131 PROM text spans identified across 64,027 notes (17.0% of the full corpus), a prevalence consistent with that of human annotation in the initial annotation training set (53 of 300 notes, 17.7%). PROM documentation was identified in 13.8% (n=32,341) of other intermediate visits, 32.7% (n=18,519) of first (consult) visits, 13.2% (n=2365) of visits preceding a 60-day gap in care, 13.9% (n=1733) of visits following a 60-day gap in care, and 16.0% (n=9069) final visits within 1 year. The prevalence of documented PROM use, by visit, decreased over time (19.1% in FY2018, 16.5% in FY2019, and 13.2% in FY2020) (Multimedia Appendix 3). The most documented PROMs were the Bournemouth Questionnaire (back and neck versions) and the Oswestry Disability Index.

Rule-based model performance across the strict- and soft-boundary matching and note categorization tasks are shown in Table 2. Performance for the strict-boundary matching task was low across all metrics. When relaxing the matching criteria to allow for soft-boundary overlap, the model performance improved substantially with good to excellent model performance that was balanced across precision and recall with few false positives and very few false negatives. Rule-based model performance in the note categorization task was excellent, with high precision and near-perfect recall.

**Figure 4.**
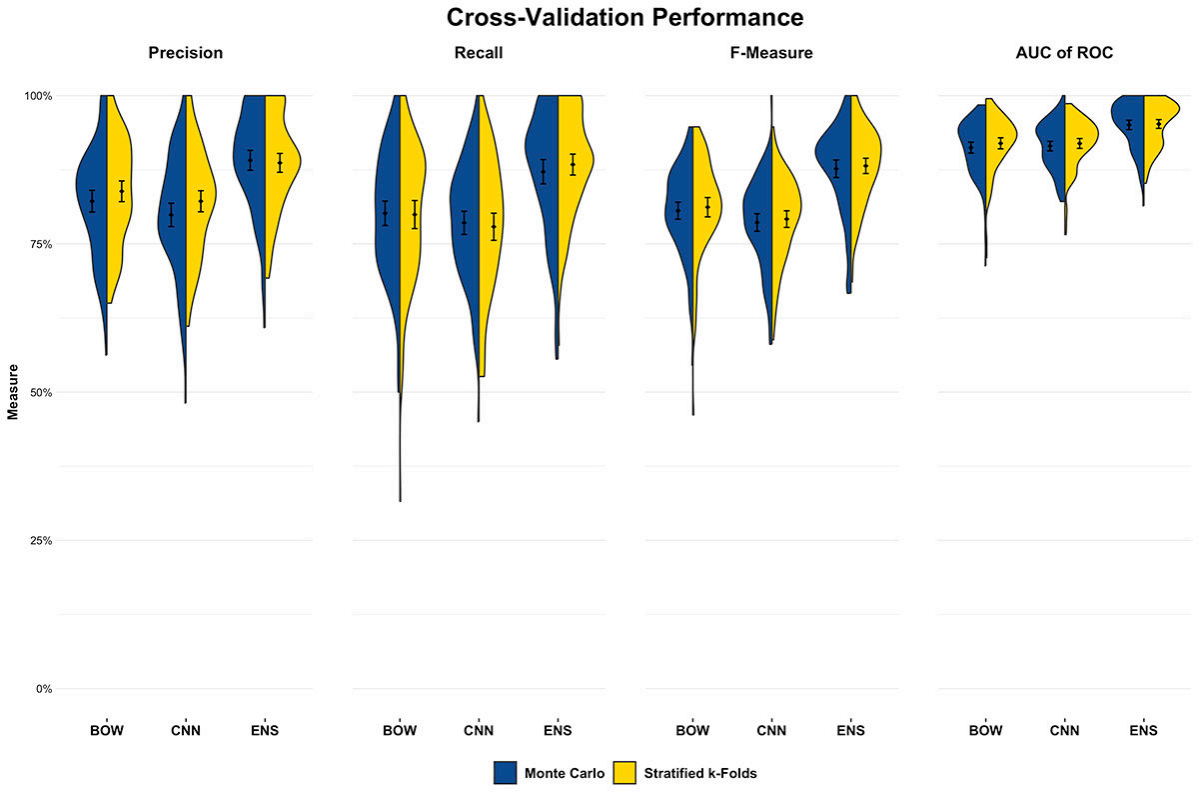
Performance metric distributions (with mean value and 95% CIs) for the note text categorization task using Monte Carlo and stratified k-folds cross-validation for the bag-of-words (BOW), convoluted neural network (CNN), and ensemble (ENS) models. AUC of ROC: area under the receiver operator characteristic curve.

The comparison between the rule-based model and the initial statistical and machine learning model performance on the note categorization task is shown in Table 3. Using the rule-based model output as a binary text categorization yielded better performance across all metrics and high accuracy (95.8%) compared with the spaCy models. Performance metric distributions (Figure 4) and the mean metric with 95% CI (Table 4) were consistent across each cross-validation method, with acceptable to good model performance across all metrics for all spaCy models. The model consistently outperformed both the BOW and convoluted neural network models across all metrics, with a good balance between precision and recall and a high AUC-ROC.

**Table 1.** Patient and visit sample characteristics.

Characteristics	Total
Patient characteristics	
Total patients, n	56,628
Age^a^ (years), median (IQR)	53 (39-66)
Sex, n (%)	
Female	9119 (16.1)
Male	47,509 (83.9)
Race or ethnicity, n (%)	
White	38,921 (68.7)
Black or African American	9899 (17.5)
Hispanic or Latino	4410 (7.8)
Other or unknown	3398 (6.0)
Marital status, n (%)	
Married	29,653 (52.4)
Single or never married	9456 (16.7)
Divorce or separated	15,650 (27.6)
Other or unknown	1869 (3.3)
Smoking status, n (%)	
Current smoker	19,133 (33.8)
Former smoker	15,872 (28.0)
Never smoker	21,571 (38.1)
Missing	52 (0.1)
Service-connected percentage, median (IQR)	70.0 (10.0-90.0)
BMI^b^ (kg/m^2^), median (IQR)	29.4 (26.2-33.1)
Visit characteristics	
Total visits and notes, n	377,213
Note token length, median (IQR)	447 (261-718)
Chiropractic care visits per patient, median (IQR)	5.0 (3.0-9.0)
Visit diagnoses, median (IQR)	
Low back pain visits	4.0 (2.0-7.0)
Neck pain visits	1.0 (0.0-5.0)
Other spinal pain visits	0.0 (0.0-4.0)
Other diagnosis visits	2.0 (0.0-5.0)
Visit types, n (%)	
First (consult) visit	56,628 (15.0)
Final visit within 1 year	56,628 (15.0)
Visits preceding a 60-day care gap	17,890 (4.7)
Visits following a 60-day care gap	12,466 (3.3)
Other intermediate visit	233,601 (62.0)

a Age as of initial chiropractic consult.

b 7108 missing.

**Table 2.** Rule-based model evaluation summary.

Rule-based model task	Precision (%)	Recall (%)	F-measure (%)
Strict-boundary matching task	47.4	58.0	52.2
Soft-boundary matching task	81.1	96.7	88.2
Note categorization task	90.3	99.5	94.7

**Table 3.** Note categorization task model evaluation summary.

Model	Precision (%)	Recall (%)	F-measure (%)	Accuracy (%)	AUC-ROC^a^ (%)
Rule-based model	90.3	99.5	94.7	95.8	—^b^
Bag-of-words model	75.0	85.7	80.0	82.0	93.8
Convoluted neural network model	93.8	71.4	81.1	86.0	94.7
Ensemble model	86.4	90.5	88.4	90.0	96.5

a AUC-ROC: area under the receiver operator characteristic curve.

b Not applicable.

**Table 4.** Statistical and machine learning model performance metrics from Monte Carlo (100 iterations) and k-folds (10x10-folds) cross-validation (stratified and unstratified).

	Precision (%), mean (95% CI)	Recall (%), mean (95% CI)	F-measure (%), mean (95% CI)	AUC-ROC^a^ (%), mean (95% CI)
Monte Carlo cross-validation				
Bag-of-words	82.2 (80.4-84.0)	80.2 (78.1-82.2)	80.6 (79.2-82.0)	91.2 (90.3-92.2)
Convoluted neural network	79.9 (77.9-81.9)	78.5 (76.6-80.5)	78.6 (77.1-80.1)	91.5 (90.7-92.3)
Ensemble	89.1 (87.4-90.8)	87.2 (85.1-89.2)	87.7 (86.2-89.2)	95.1 (94.3-95.9)
k-folds cross-validation				
Bag-of-words	81.3 (79.5-83.2)	82.1 (80.0-84.2)	81.1 (79.6-82.6)	92.2 (91.4-92.9)
Convoluted neural network	79.6 (77.5-81.7)	79.5 (77.4-81.7)	78.9 (77.2-80.5)	92.2 (91.5-93.0)
Ensemble	88.7 (87.1-90.3)	87.2 (85.4-89.1)	87.6 (86.1-89.0)	95.0 (94.1-95.8)
Stratified k-folds cross-validation				
Bag-of-words	83.9 (82.1-85.6)	79.9 (77.6-82.3)	81.2 (79.6-82.8)	92.0 (91.0-92.9)
Convoluted neural network	82.2 (80.4-84.0)	77.9 (75.6-80.2)	79.2 (77.8-80.6)	91.9 (91.1-92.8)
Ensemble	88.7 (87.1-90.3)	88.4 (86.6-90.1)	88.2 (86.9-89.4)	95.2 (94.5-96.0)

a AUC-ROC: area under the receiver operator characteristic curve.

## Discussion

### Principal Findings

In this study, we tested NLP approaches to identify PROM use documented in VHA chiropractic clinic notes as a method to monitor an important marker of high-quality chiropractic care. Our iterative process targeting a series of prediction tasks showed overall strong performance across rule-based, statistical, and machine-learning approaches, affirmed by multiple approaches to cross-validation. The rule-based NLP model had good to excellent performance in identifying text spans referencing PROMs when flexible span boundaries were used but not when span boundaries were strict. When using the rule-based model output as a note categorization prediction, overall performance was excellent, with high precision and near-perfect recall. While our initial hypothesis predicted comparable performance between the rule-based approach and the statistical and machine learning methods, our findings demonstrate that the rule-based approach achieved superior performance in identifying and categorizing PROM documentation within this sample of VHA chiropractic clinic text notes. We suspect improved performance may be attributable to data volume and sparsity of text indicators of PROMs relative to the remaining document content describing the rest of the chiropractic care encounter. Rule-based models are generally less susceptible to data volume issues given their methodical approach, whereas machine learning approaches thrive on large data sets where more complex patterns and relationships can be trained on and identified in the text. Important benefits of the rule-based approach include simplicity, interpretability, and efficiency—all of which provide added value alongside slightly superior performance.

Overall model performance was sufficient to have clinical utility for quality metric monitoring, especially given the low prevalence of false negatives. The error analysis process used during model development identified that remaining false positives were frequently due to unique circumstances difficult to address by creating exclusion rules without overfitting the training data. For example, the text “Goals of Care: Improve outcome measures, reduce ODI by 20%” would identify a section span to exclude (“Goals of Care”) followed by a section span to include (“outcome measures”) and a PROM span (“ODI”), therefore marking the PROM span for inclusion.

Consistent findings across multiple methods of cross-validation, with and without stratification accounting for baseline PROM use prevalence, showed acceptable performance for each of the statistical and machine learning models tested on the note categorization task. Of note, several performance metrics in the initial model evaluation fell outside the cross-validation estimated 95% CI, attesting to the benefit of cross-validation in generating a more appropriate representation of performance.

### Clinical Implications and Future Work

While all models performed adequately for note categorization, we used the rule-based model, having the best performance, on the full corpus to estimate the overall prevalence of PROM documentation. The overall prevalence was low and consistent with estimates from the annotation data, highlighting the potential limited documented use of PROMs by VHA chiropractors despite being an identified quality care metric. This is in contrast with data from surveys of US chiropractors in which 60% of respondents reported using PROMs several times per day [27]. Qualitative evaluation in a non-VHA setting has highlighted multiple barriers and facilitators to implementing PROMs in chiropractic care [28]. These included clinician knowledge and training, engagement and purpose of collecting PROM data, perceived utility versus burden for clinicians and patients, and organizational and administrative factors (such as in-visit time availability and ability to use electronic data collection systems). Evidence of external determinants influencing PROM use can be hypothesized from our results showing a decreasing trend in documented PROM use across each year of the study period. The VHA Chiropractic Program rapidly expanded nationally during this time, with an increase in the number of employed VHA chiropractors [29] and increasing service penetration across the national VHA system [30]. Thus it is possible that our results reflect an increasing number of new chiropractors not using PROMs, not documenting PROM use in clinic notes, or a combined effect of the 2, highlighting a potential opportunity for quality improvement and education.

Given our findings, we hypothesize there is a relationship between documentation quantity as a proxy for comprehensiveness of evaluation, including the use of PROMs. PROM use was most prevalent in first (consult) visits at more than double the rate found in other visit types. Paired with significantly longer text lengths compared with other visit types, this suggests that the increased complexity and information gathering inherent in initial consultations necessitate more thorough documentation, including the application of outcome measures. Additionally, the decrease in documented PROM use during FY2020, paired with shorter text length in FY2020, is potentially attributable to the onset of the COVID-19 pandemic with changes in the quantity and mode of delivery of chiropractic care [31] and challenges in administration due to impacted face-to-face care with limited availability of remote data collection information systems.

Future work should consider patient, visit, facility, clinician, and system factors, including qualitative perspectives of VHA chiropractors, that may influence the use of specific PROMs and their documentation by VHA chiropractors. These determinants can inform intervention development and implementation strategies related to improving PROM use and documentation. However, these potential external influences do not affect our confidence in the performance of the developed NLP models to evaluate documented PROM use by VHA chiropractors on a national scale as a quality metric.

The implications of tracking PROM use by VHA chiropractors using this approach include enhanced patient outcomes, improved clinical decision-making, and ensuring consistent, high-quality chiropractic care for veterans. These are consistent with core practical considerations for the development of NLP systems to address pragmatic clinical needs and improve patient outcomes, including offering an organization incentive for use and supporting ongoing monitoring with implementation feasibility [32]. Within the VHA chiropractic care setting, our approach allows for the monitoring of an important quality metric at a national scale using centralized resources. Additionally, this approach standardizes and enhances the objectivity and rigor of assessment of documentation content, while minimizing the individual burden of chart review by practicing VHA chiropractors. Further, our methods may have application in other clinical settings providing musculoskeletal pain care, in the VHA and beyond, given the high prevalence of musculoskeletal pain in both veteran and nonveteran populations. Future research should focus on refining these NLP models to enhance their applicability across diverse clinical settings, including other pain care clinics, and explore the integration of additional data sources to further enrich patient care insights. Additionally, future research can evaluate the efficiency of our approach in terms of financial, human, computational, and other resource costs compared with traditional manual review methods to best understand the potential value and system resource use implications of this work.

### Limitations

There are several limitations to our study that are inherent to any observational research or studies using NLP. The text notes used in this study were originally intended for clinical care purposes. Thus, their secondary use for research is subject to limitations in the quality of the notes and the content documented for that purpose, which may not always fully reflect what was done during the patient encounter. Observing the variation in the quantity of text (ie, number of tokens) present in each note across the corpus showed an expectedly right-skewed distribution and highlighted heterogeneity in the quantity of text content included in VHA chiropractic clinic notes. Similar findings were evident in tokenized length differences between visit types and the year during which a visit occurred. We suspect this correlates with heterogeneity in comprehensiveness of documentation, and by proxy of care delivered, highlighting a future opportunity for VHA chiropractor education or other quality improvement interventions to ensure consistent quality on a national scale. Templated material in clinic notes, while difficult to quantify across the corpus, may limit the richness and variability of the text data, potentially affecting the capture of patient- and note-specific details. It was evident during annotation that templates were, at times, shared across VHA facilities, which may affect our intention to capture variability across the VHA by stratifying our random sampling of notes by the facility. Further, our sampling by facility may not account for variations between individual chiropractors at a given facility. Stratifying our random sampling by a chiropractor was an alternative strategy but risked increasing the heterogeneity of the sample.

Our strategy to capture clinic notes is consistent with previous practices to identify VHA chiropractic clinic visits and associated notes. However, facility variation in clinic workflow may also influence the use and the documentation of PROMs separately. While expected that these would be documented in the EHR, clinics may use alternative strategies for the collection and recording of PROMs from patients and limiting our findings to representing “documented PROM use.” Further, the initial seed list of PROMs suspected to potentially be used by VHA chiropractors was established a priori without empirical validation. We allowed flexible expansion of the initial list during the annotation process to include PROMs that had use, but were not considered in advance, yet it is possible we still failed to include PROMs that may have been used more rarely than our random sampling could capture. As future PROMs are developed and adopted, maintenance of the proposed NLP approach to incorporate these has not been assessed in this study. While this would require some degree of manual effort, we do not suspect that incorporating additional PROMs into the model is particularly challenging given our experience in expanding the initial seed list during this study. We also did not incorporate pretrained or large-language models into this analysis due to restricted use in our computing environment at the time of this study. If, in future research, these types of models are successfully able to be validated with adequate performance and implemented, their adoption may mitigate the requirement of ongoing manual efforts. We also did not conduct a formal text feature analysis on the output from the tested machine learning models, which could provide insight into the contributions of specific text to the model prediction. This may be an important contribution in future work to compare the explainability of the machine learning approaches to the generally interpretable rule-based approach and potentially optimize machine learning model parameters to enhance prediction performance.

Our sample of patients receiving VHA chiropractic care from the MSD/CIH Cohort allowed overlapping entry into the parent cohort and our study sample through the end of the study period, ideally representing all chiropractic care occurring during this time. However, by limiting our follow-up period for an individual patient to 1 year after their initial chiropractic visit, we may have excluded chiropractic care received later in the study period. This, along with the onset of the COVID-19 pandemic, may account for the rapid reduction in patients and visits during the final year of the cohort with unknown potential impact on our findings.

As all notes in this study originated during VHA chiropractic care, there is unknown utility of the developed NLP models in non-VHA chiropractic documentation. Variations in the documentation requirements between the VHA and non-VHA settings for administrative purposes (eg, billing) may influence the use and documentation of PROMs. Nonetheless, our sample originates from the largest collection of chiropractic care EHR data in an integrated medical setting, with evidence from VHA studies of chiropractic care having the potential to influence chiropractic care in the non-VHA setting.

### Conclusions

Our study demonstrates the effective use of NLP to accurately identify documented PROM use from VHA chiropractic clinic notes, highlighting the potential for improved data use in quality monitoring of patient care. By leveraging an NLP approach, we can overcome the challenges posed by unstructured clinical text notes to track an identified quality care metric for chiropractic care. Overall documented use of PROMs was low and highlights the need for quality improvement. Future work should evaluate determinants influencing PROM use and develop intervention and implementation strategies to improve their use and documentation in VHA chiropractic care to ensure consistent, high-quality chiropractic care for veterans.

## Supplementary material

10.2196/66466 Multimedia Appendix 1 Patient-reported outcome measure (PROM) span match and note frequencies in annotation set and full text corpus.

10.2196/66466 Multimedia Appendix 2 Supplemental methods describing detailed architectures of statistical and machine learning models.

10.2196/66466 Multimedia Appendix 3 Trends in patient-reported outcome measure use, by unique patient and visit, across the study period, by fiscal year (FY) and month. Patient-reported outcome measure use (PROM +) identified based on note (visit) categorization output from the rule-based natural language processing (NLP) model. Individual patients may be counted in multiple months and fiscal years.

## Abbreviations

AUC-ROC: area under the receiver operator characteristic curve
BOW: bag-of-words
EHR: electronic health record
FY: fiscal year
IAA: interannotator agreement
*ICD-10*: *International Classification of Diseases, 10th Revision*
MSD/CIH: Musculoskeletal Diagnosis/Complementary and Integrative Health
NLP: natural language processing
PROM: patient-reported outcome measure
VHA: Veterans Health Administration
