# Supplementary material for: Identifying Patient-Reported Outcome Measure Documentation in Veterans Health Administration Chiropractic Clinic Notes: Natural Language Processing Analysis
Source: JMIR Med Inform. 2025 Apr 2;13:e66466. doi: 10.2196/66466 (PMC12038758; doi:10.2196/66466)
Supplement: Multimedia Appendix 1 [file medinform-v13-e66466-s001.docx]

**Multimedia Appendix 1.** Patient-reported outcome measure (PROM) span match and note frequencies in annotation set and full text corpus.

|  | **Annotation Set (n=500 notes)** | | | | |  | **Full Text Corpus (n=377,213 notes)** | | |
| --- | --- | --- | --- | --- | --- | --- | --- | --- | --- |
|  | **Human Annotation** | |  | **Rule-Based Model** | |  | **Rule-Based Model** | | |
| **Patient Reported Outcome Measure** | **Span Frequency (n)** | **Note Frequency (n)** |  | **Span**  **Frequency (n)** | **Note**  **Frequency (n)** |  | **Span**  **Frequency (n)** |  | **Note**  **Frequency (n)** |
| Bournemouth (Neck and Back versions) | 65 | 53 |  | 77 | 53 |  | 30,619 |  | 20,425 |
| Oswestry Disability Index | 61 | 54 |  | 75 | 63 |  | 24,123 |  | 20,366 |
| Neck Disability Index* | 38 | 34 |  | 44 | 34 |  | 13,863 |  | 11,258 |
| PROMIS Measures (e.g., PROMIS 6b) | 20 | 18 |  | 21 | 17 |  | 8,729 |  | 5,925 |
| Defense and Veterans Pain Rating Scale (DVPRS) | 17 | 17 |  | 17 | 17 |  | 6,993 |  | 6,796 |
| Pain Disability Questionnaire | 16 | 9 |  | 25 | 10 |  | 4,964 |  | 2,148 |
| Functional Rating Index* | 15 | 10 |  | 17 | 11 |  | 2,642 |  | 1,726 |
| Keele STarT Back Screening Tool | 14 | 14 |  | 10 | 10 |  | 1,998 |  | 1,935 |
| Pain Global Rating of Change | 12 | 11 |  | 21 | 14 |  | 8,636 |  | 3,801 |
| Pain, Enjoyment of Life, and General Activity (PEG-3) Scale | 12 | 12 |  | 13 | 13 |  | 3,052 |  | 2,956 |
| Tampa Kinesiophobia Scale* | 11 | 11 |  | 12 | 12 |  | 3,215 |  | 3,202 |
| Patient Specific Functional Scale | 5 | 4 |  | 7 | 4 |  | 2,229 |  | 1,197 |
| Brief Pain Inventory | 4 | 4 |  | 4 | 4 |  | 62 |  | 58 |
| Roland Morris Disability Questionnaire | 3 | 3 |  | 3 | 3 |  | 239 |  | 173 |
| Pain Disability Index | 0 | 0 |  | 4 | 4 |  | 661 |  | 622 |
| Chronic Pain Grade Questionnaire | 0 | 0 |  | 3 | 3 |  | 22 |  | 22 |
| Multidimensional Pain Inventory | 0 | 0 |  | 3 | 3 |  | 48 |  | 44 |
| Short-Form Surveys (e.g., SF-36) | 0 | 0 |  | 3 | 3 |  | 36 |  | 36 |

**Indicates PROM added in second iteration based on review of initial annotation*
