# Supplementary material for: Identifying Patient-Reported Outcome Measure Documentation in Veterans Health Administration Chiropractic Clinic Notes: Natural Language Processing Analysis
Source: JMIR Med Inform. 2025 Apr 2;13:e66466. doi: 10.2196/66466 (PMC12038758; doi:10.2196/66466)
Supplement: Multimedia Appendix 2 [file medinform-v13-e66466-s002.docx]

**Multimedia Appendix 2.** Supplemental methods describing detailed architectures of statistical and machine learning models.

In this study, three spaCy models were trained and tested for performance in the note categorization (i.e., text categorization) task: 1) a bag-of-words model, 2) a convoluted neural network model, and 3) an ensemble model combining a linear bag-of-words model and Tok2Vec model. Configuration files are available on GitHub, with summary details described briefly below.

**Bag of Words Model**

This spaCy configuration defines a text classification model that employs a bag-of-words approach, representing text as a collection of English word occurrences without considering their order. The model uses a linear classifier trained on the corpus, where each word in the vocabulary is assigned a weight. During prediction, the model calculates a weighted sum of word occurrences for each class (“positive” or “negative”) and predicts the class with the highest score. This configuration specifies a single-word n-gram size, exclusive class assignments, and a training approach with Adam optimization, dropout regularization, and early stopping based on performance on a held-out development set. The model is designed for efficient batch processing and incorporates features like vocabulary loading and pre-trained word embeddings for improved performance.

**Convoluted Neural Network Model**This spaCy configuration defines a text classification model that uses a Convolutional Neural Network (CNN) architecture, which can (to an extent) incorporate word order and context. The model employs embeddings to represent words numerically and then uses convolutional filters to extract features from sequences of word embeddings. These features are then fed into a classifier to predict the text category. This allows the model to capture local and global contextual information, unlike simpler models that treat words independently. The specific CNN architecture in this configuration employs character-level hashing for word embeddings (HashEmbedCNN), which is efficient and memory-friendly compared to traditional word embeddings. This is followed by four convolutional layers to extract features from character sequences at different levels of abstraction and an implicit softmax layer to convert the output vector into a probability and prediction of the involved classes. This approach enables the model to learn complex relationships between characters and words, improving its ability to understand and classify text effectively. This CNN architecture is well-suited for text classification tasks where character-level information can be crucial for capturing subtle linguistic nuances and handling out-of-vocabulary words effectively. The model is trained using standard techniques such as dropout regularization and Adam optimization, with early stopping to prevent overfitting.

**Ensemble Model**

The Ensemble model defines a text classification model that combines multiple machine learning models into a single functional system with the goal of improving overall performance. This model employs a combination of a bag-of-words model, a word embedding model, and a window encoder to represent the text data and ideally achieve better classification accuracy. The TextCatBOW component represents text as the collection of word frequencies, considering n-grams of length 1 (i.e., individual tokens). The Ensemble architecture also uses a Tok2Vec component to encompass a word embedding and encoding approach to create a numerical vector representation of the text with multiple embedding layers to represent text meaning. This includes the MultiHashEmbed layer to map features to dense vectors (semantic relationships) and the MaxoutWindowEncoder to combine word vectors from a window to consider contextual information. The outputs from the TextCatBOW and the Tok2Vec components are integrated within the Ensemble model to create a robust representation of the input text for the text classification task.
