# Supplementary material for: Identifying Patient-Reported Outcome Measure Documentation in Veterans Health Administration Chiropractic Clinic Notes: Natural Language Processing Analysis
Source: JMIR Med Inform. 2025 Apr 2;13:e66466. doi: 10.2196/66466 (PMC12038758; doi:10.2196/66466)
Supplement: Multimedia Appendix 3 [file medinform-v13-e66466-s003.docx]

**Multimedia Appendix 3.** Trends in patient reported outcome measure use, by unique patient and visit, across the study period, by fiscal year (FY) and month. Patient reported outcome measure use (PROM +) identified based on note (visit) categorization output from the rule-based NLP model. Individual patients may be counted in multiple months and fiscal years.

|  | **Patients** | | |  | **Visits** | | |
| --- | --- | --- | --- | --- | --- | --- | --- |
| **Date** | **Total (N)** | **PROM + (N)** | **Prevalence (%)** |  | **Total (N)** | **PROM + (N)** | **Prevalence (%)** |
| **FY2018** | **26,078** | **8,951** | **34.3** |  | **130,146** | **24,842** | **19.1** |
| Oct 2017 | 2,205 | 735 | 33.3 |  | 3,463 | 823 | 23.8 |
| Nov 2017 | 3,638 | 928 | 25.5 |  | 6,172 | 1,218 | 19.7 |
| Dec 2017 | 4,586 | 1,092 | 23.8 |  | 7,152 | 1,438 | 20.1 |
| Jan 2018 | 5,870 | 1,452 | 24.7 |  | 9,727 | 2,125 | 21.8 |
| Feb 2018 | 6,145 | 1,399 | 22.8 |  | 9,775 | 1,942 | 19.9 |
| Mar 2018 | 7,107 | 1,594 | 22.4 |  | 11,429 | 2,293 | 20.1 |
| Apr 2018 | 7,607 | 1,773 | 23.3 |  | 12,135 | 2,539 | 20.9 |
| May 2018 | 8,253 | 1,811 | 21.9 |  | 13,394 | 2,639 | 19.7 |
| Jun 2018 | 8,386 | 1,831 | 21.8 |  | 13,114 | 2,560 | 19.5 |
| Jul 2018 | 8,372 | 1,666 | 19.9 |  | 13,070 | 2,264 | 17.3 |
| Aug 2018 | 9,747 | 1,885 | 19.3 |  | 16,092 | 2,648 | 16.5 |
| Sep 2018 | 9,545 | 1,717 | 18.0 |  | 14,623 | 2,353 | 16.1 |
| **FY2019** | **41,844** | **13,152** | **31.4** |  | **200,860** | **33,101** | **16.5** |
| Oct 2018 | 10,884 | 2,140 | 19.7 |  | 17,714 | 2,967 | 16.7 |
| Nov 2018 | 10,050 | 1,909 | 19.0 |  | 15,408 | 2,541 | 16.5 |
| Dec 2018 | 9,240 | 1,738 | 18.8 |  | 13,473 | 2,130 | 15.8 |
| Jan 2019 | 10,811 | 2,102 | 19.4 |  | 17,120 | 2,796 | 16.3 |
| Feb 2019 | 10,079 | 1,943 | 19.3 |  | 15,097 | 2,508 | 16.6 |
| Mar 2019 | 10,578 | 2,132 | 20.2 |  | 15,965 | 2,747 | 17.2 |
| Apr 2019 | 11,446 | 2,269 | 19.8 |  | 18,198 | 2,895 | 15.9 |
| May 2019 | 11,740 | 2,263 | 19.3 |  | 18,353 | 2,952 | 16.1 |
| Jun 2019 | 11,126 | 2,165 | 19.5 |  | 16,467 | 2,770 | 16.8 |
| Jul 2019 | 11,412 | 2,238 | 19.6 |  | 17,504 | 2,867 | 16.4 |
| Aug 2019 | 11,905 | 2,266 | 19.0 |  | 18,325 | 3,000 | 16.4 |
| Sep 2019 | 11,602 | 2,272 | 19.6 |  | 17,236 | 2,928 | 17.0 |
| **FY2020** | **12,708** | **2,402** | **18.9** |  | **46,207** | **6,084** | **13.2** |
| Oct 2019 | 9,248 | 1,443 | 15.6 |  | 14,356 | 2,043 | 14.2 |
| Nov 2019 | 6,500 | 995 | 15.3 |  | 9,078 | 1,271 | 14.0 |
| Dec 2019 | 5,121 | 689 | 13.5 |  | 6,766 | 834 | 12.3 |
| Jan 2020 | 4,686 | 638 | 13.6 |  | 6,363 | 794 | 12.5 |
| Feb 2020 | 3,584 | 482 | 13.4 |  | 4,672 | 592 | 12.7 |
| Mar 2020 | 2,334 | 303 | 13.0 |  | 2,725 | 328 | 12.0 |
| Apr 2020 | 340 | 6 | 1.8 |  | 399 | 6 | 1.5 |
| May 2020 | 253 | 18 | 7.1 |  | 321 | 19 | 5.9 |
| Jun 2020 | 393 | 51 | 13.0 |  | 508 | 59 | 11.6 |
| Jul 2020 | 416 | 54 | 13.0 |  | 529 | 63 | 11.9 |
| Aug 2020 | 284 | 46 | 16.2 |  | 341 | 58 | 17.0 |
| Sep 2020 | 120 | 14 | 11.7 |  | 149 | 17 | 11.4 |
